# Analysing the progress in service delivery towards achieving universal health coverage in Nigeria: a scoping review

**DOI:** 10.1186/s12913-023-10090-w

**Published:** 2023-10-12

**Authors:** Chinyere Okeke, Uchenna Ezenwaka, Adanma Ekenna, Chioma Onyedinma, Obinna Onwujekwe

**Affiliations:** 1 Health Policy Research Group, University of Nigeria Enugu Campus, Enugu, Nigeria; 2 Department of Community Medicine, College of Medicine, University of Nigeria Enugu Campus, Enugu, Nigeria; 3 Department of Health Administration and Management, Faculty of Health Sciences and Technology, University of Nigeria Enugu Campus, Enugu, Nigeria

**Keywords:** Coverage, Equity, Health Service Delivery, Nigeria, Universal Health Coverage, UHC

## Abstract

**Background:**

Attainment of universal health coverage (UHC) requires optimal utilization of health services. Poor coverage and inequitable access to healthcare could hinder improvement in service delivery towards UHC. The study analyzed the progress in service delivery coverage and equity in access to care within the Nigerian health systems based on the tracer indicators of the WHO framework for monitoring UHC.

**Methods:**

We searched the literature in databases: PubMed, Scopus, Directory of Open Access Journals, Google Scholar, Science Direct and websites of relevant health Ministries, Agencies, and Organizations between March to December 2022. Search terms were identified in four broader themes: Service delivery coverage, equity, UHC and Nigeria. Data were collected through a review of 37 published articles (19 peer-reviewed articles and 8 grey documents). We synthesized the findings in thematic areas using the WHO framework for monitoring UHC.

**Results:**

The findings show a slow improvement in service delivery coverage across the UHC tracer indicators; reproductive, maternal, newborn and child health, infectious diseases, non-communicable diseases and service capacity and access. With regards to equity in access to care across the tracer indicators, there has been a great disparity in the utilization of healthcare services among rural dwellers, lower educational level individuals and those with poor socio-economic status over 20 years. However, there was remarkable progress in the ownership and use of long-lasting insecticide-treated nets among rural and lowest-wealth quantile households than their urban counterpart.

**Conclusion:**

There is poor coverage and persistent inequitable access to care among the tracer indicators for monitoring progress in service delivery. Attaining UHC requires concerted efforts and investment of more resources in service delivery to address inequitable access to care and sustainable service coverage for improved health outcomes.

## Background

Service delivery is widely recognized as a backbone of the health system and a functional service delivery system is one that provides quality, equitable, affordable, and accessible care [1]. The World Health Organization (WHO) defined service delivery as the way inputs are combined to allow the delivery of a series of interventions/health actions through multiple actors in the public and private sectors [2].

Appropriate and top-quality service delivery is one of the major means of the achievement of Universal Health Coverage (UHC). UHC means all people receiving the health services they need, including health initiatives designed to promote better health prevent illness, and provide treatment, rehabilitation, and palliative care of sufficient quality to be effective while at the same time ensuring that the use of these services does not expose the user to financial hardship [3].

Attaining UHC is a priority for the global health agenda, particularly in developing countries including Nigeria. More so, the 2030 Agenda for Sustainable Development Goals (SDGs) has included commitments by African Governments to expand access to health services to improve population health outcomes [4]. The SDG goal 3.8.1 seeks to improve coverage of essential health services, defined as the average coverage of essential services based on tracer indicators interventions that include reproductive, maternal, new-born and child health, infectious diseases, non-communicable diseases and service capacity and access, among the general and the most disadvantaged population [5].

Nigeria’s health system has been struggling to be on sure feet for achieving UHC and progress towards this goal has unfolded at a rather slow pace. Nigeria is placed at 142 out of 195 countries according to a Lancet report´s ranking of health systems performance using healthcare access and quality as its criteria [1]. Nigeria also ranks poorly based on the World Bank's Universal Health Coverage Service Coverage Index [2]. Evidence shows that there has been a lack of political commitment, necessitating serious health investments [6]. In most African countries, the major constraining factor to optimal service delivery includes weak and poor coordination at all levels of care, fragmented services, dearth of financial resources and non-financial resources (including drugs and supplies, inadequate and decaying infrastructure), skewed distribution of available quality of care and inequitable access to health services [7]. In addition, geographical and financial access to care, the disparity in services among socioeconomic status, and high out-of-pocket expenditure for healthcare remain a challenge facing the Nigerian health system [8, 9].

In recognition of the poor coverage and access to care and towards achieving UHC, there are recommendations to improve UHC and to monitor the progress toward achieving this [3]. Nigeria developed and has been implementing several health policies and guidelines such as the National Health Act, National Strategic Health Development Plans, Quality of Care Guideline for Primary Health Care for describing standards for health service delivery to ensure improved coverage of health service delivery [10]. However, there still exist wide gaps in service coverage tracer indicators which are worsened by inequity in access to care [10]. Following member states’ agreement to achieve UHC, it, therefore, became imperative to analyze Nigeria’s progress towards the achievement of UHC and SDG 3.8 goal [7, 11, 12].

This paper provides new information on achievements in service delivery coverage and equity in access to healthcare within the Nigerian health systems based on the tracer indicators of the WHO framework for monitoring UHC. It highlights the (dis)parities in the health service delivery system with regards to equity indices (including socio-economic status, geographic location, and educational level) influence on service delivery improvement towards the attainment of UHC. It uses a mix of different sources of the latest local data (MICS and NDHS) to show the progress made. The study will be beneficial to politicians, policymakers across health parastatals, and program managers/ implementers in planning and mobilizing resources for service delivery.

## Methods

### Study framework

**Fig. 1:**
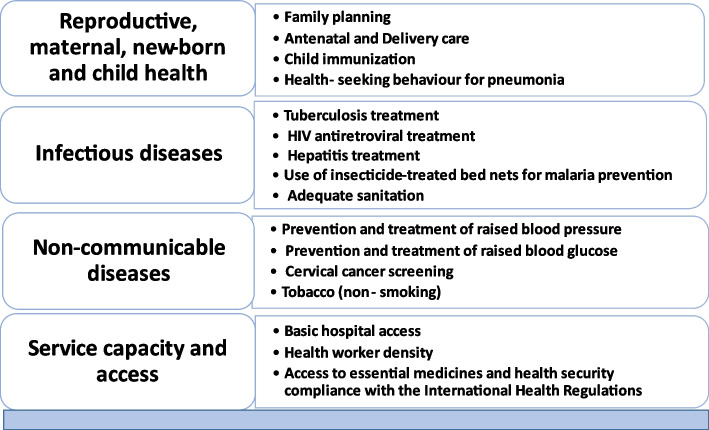
WHO service delivery indicators for monitoring UHC (WHO, 2019)

To analyze the service delivery progress of the Nigerian health system towards achieving UHC, the WHO framework for assessing coverage of service delivery toward achieving UHC was used as a guide [13]. The framework listed 16 key health services tracer indicators and was grouped into four subsections. The sub-sections include reproductive, maternal, newborn, and child health (RMNCH); infectious diseases; non-communicable diseases; and service capacity and access. Figure 1 shows the 16 WHO tracers indicators for monitoring UHC service delivery grouped under 4 main subsections.

### Study design and setting

We undertook a scoping review of grey and published literature on service delivery outcomes in Nigeria to identify progress in service delivery coverage and equity in Nigeria. The review was deemed an appropriate research design for this study because of the paucity of analytical reviews on service delivery based on UHC tracer indicators. Our review was based on Levac et al.’s methodology which included five stages out of the six mentioned, namely identifying the research question; identifying relevant studies; selecting the studies for review; charting the data; and collating, summarizing, and reporting results [14]. The study framework as described in Fig. 1 was considered appropriate because it permits analysis of the progress of service delivery and globally accepted tracer indicators for measuring progress towards UHC.

### Data collection and search strategy

A scoping review of the literature was performed between March to December 2022. We conducted the search from several search engines and databases in health and medicine, which include Scopus, PUBMED, Directory of Open Access Journals, google scholar, and Science Direct for peer-reviewed articles published in English between 2010 and 2022 using the key words ‘service delivery’, ‘Equity’, ‘UHC’, ‘Universal Health Coverage’, ‘Nigeria’ and ‘service coverage’ AND ‘Nigeria’ in the title. The search terms ensure a systematic and uniform search was applied to the various platforms and articles were retrieved. Our search strategy was in line with Moher’s Preferred Reporting Items for Systematic Reviews and Meta-Analyses (PRISMA) guidelines [15]. In addition, we expanded our search to capture the websites of relevant Ministries, Departments, Agencies, and Organizations, such as the Ministry of Health, the World Health Organization, and the National Primary Health Care Development Agency, to identify policies and reports to ensure comprehensive coverage of all sources providing information related to service delivery and outcome in Nigeria.

### Eligibility criteria

Selected studies were eligible for review if they: (i) describe or analyze coverage of service delivery (ii) are particular about Nigeria (iii) emphasize health provision relating to reproductive, maternal and child health, communicable, non-communicable and service capacity and access (iv) are published within the 2010 to 2022 period (v) are written in the English language (vi) are of any research design, and (vii) include grey and academic literature.

### Study document selection

**Fig. 2:**
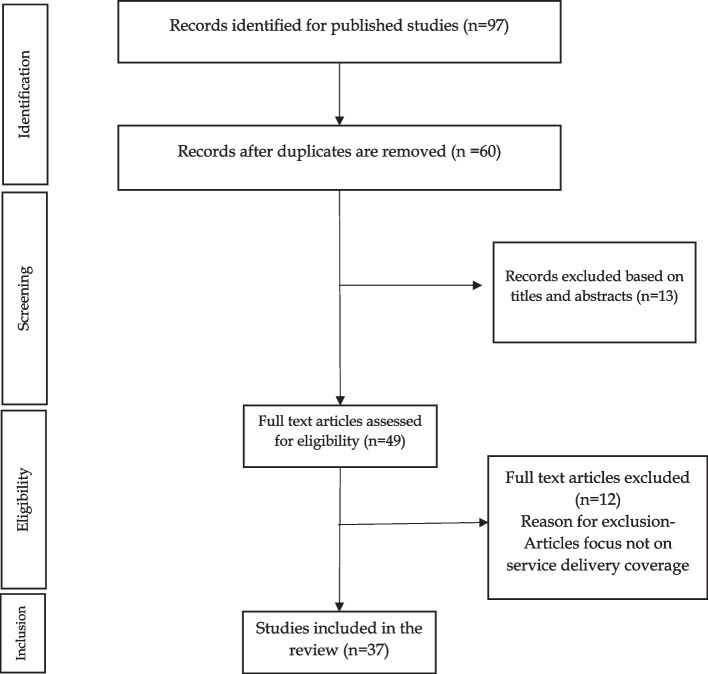
Flow chart showing detailed article extraction and evaluation method

A total of 96 published and 11 grey literature were retrieved and reviewed following our inclusion criteria and removal of duplicates. We then screened further by examining abstracts and titles, before evaluating the full contents of articles to arrive at the 37 articles (19 journal articles and 8 grey documents) reviewed in detail. The screening was done by an experienced health systems researcher with an interest in health service delivery. Disagreements that emerged during the review were either resolved by discussion or by consulting an additional reviewer. Figure 2 shows a detailed representation of the extraction and evaluation of articles.

### Data analysis

A thematic narrative synthesis was used for the data analysis. The contents of the reviewed literature were arranged based on each dimension of the study framework described above. The analysis involved extraction of information service delivery *coverage* and *equity* across WHO tracer indicators for monitoring UHC well as challenges affecting the improvement of service delivery towards attaining UHC.

## Results

The findings of the study are presented under the four sections of the WHO framework for monitoring UHC as described above.

### Coverage of health services

#### Reproductive, maternal, newborn, and child health

**Fig. 3:**
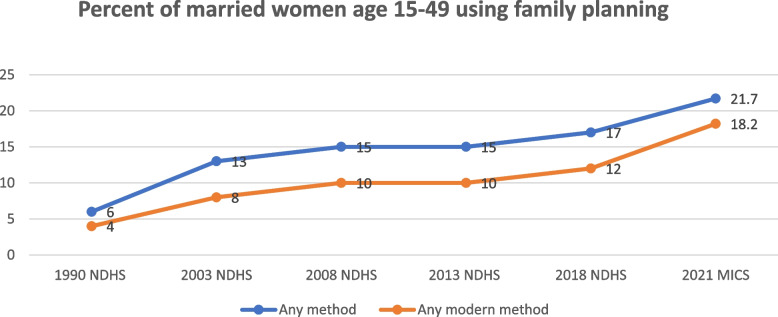
Trends in family planning utilization from 1990 to 2021 in Nigeria

Over the past twenty years, there have been slight decreases on the unmet need for modern contraception in Nigeria although it is still estimated at over 20% [4, 5]. The proportion of women whose need for family planning (FP) has been satisfied by modern methods was found to be significantly but slowly increasing through the years (Fig. 3). The National Demographic Health Survey (NDHS) [6, 7] shows a 2% increase in demand for FP from 15% in 2013 [8] to 21% in 2021 [9]. Demand for modern methods of family planning services for married women/ in-union women was 39.9% in 2021 [10].

**Fig. 4:**
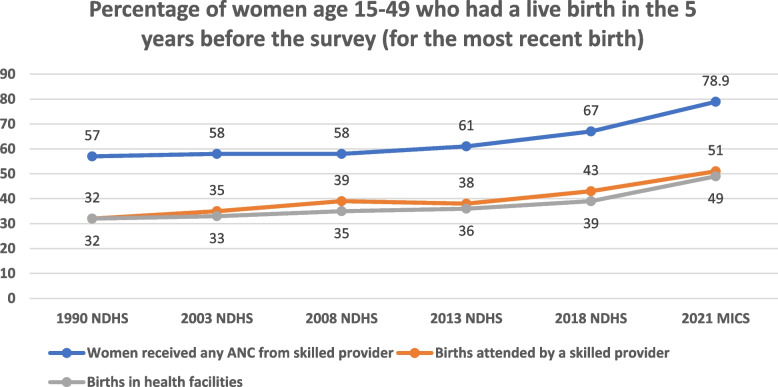
Trends in maternal health care between 1990 and 2021 in Nigeria

In the maternal health coverage area, the review found that more women utilized antenatal care services in 2021 with 21% of no attendance, 60.4% of about 4 visits and 23.2% having attended more than 8 times [10]. Findings show a 9% increase over a decade from 58% in 2008 to 67% in 2018 in the NDHS data regarding skilled care from any provider (Fig. 4). The World Bank database collating data from the Multiple Indicator Cluster Survey 2011, NDHS 2011, and the Malaria Indicator Survey 2010 and 2013 shows a similar increase from 54.1% to 62.6%. The proportion of women who are attended to by a skilled birth attendant (SBA) during delivery shows a slow increase from 32 to 43% over a decade in the NDHS report, then to 51% in 2021 (Fig. 4).

**Fig. 5:**
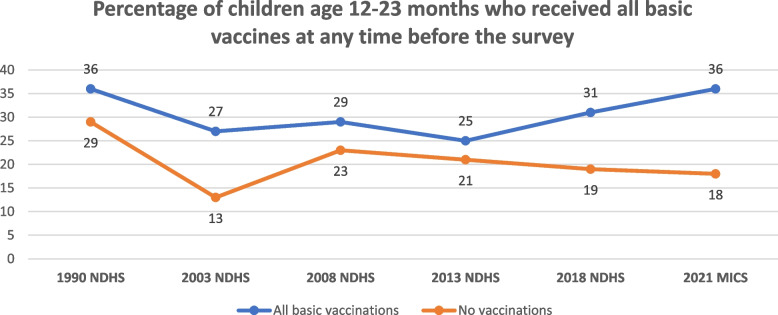
Trends in childhood vaccinations between 1990 and 2021 in Nigeria

The situation in child health indices showed a leap from 35% in 2013 [8] to 75% in 2018 [11] in the proportion of children taken to the hospital for suspected acute respiratory infections (ARIs) but reduced to 39% in 2021 [10]. However, the number of children fully immunized increased slowly from 23% in 2008 [4] to 31.3% in 2018 [11] and then to 36% in 2021 [10] (Fig. 5). Similarly, the rate of zero vaccinated children is slowly decreasing.

There has been a percentage difference in maternal mortality ratio, infant and under-five mortality of 6.2%, 27.3% and 17.3% over a decade spanning 2008 till 2018 [7].

#### Infectious disease control

There has been a slow decrease in the prevalence of Tuberculosis (TB) per 100,000 people in Nigeria from 546 in 2008 and 323 in 2018 [11, 12] with an even poorer index for tuberculosis case detection rate decrease from 19% in 2008 to 15% in 2018 [12–17]. Studies have shown a prevalence of 22.1% in 2021 [18]. The prevalence of HIV in Nigeria was at a peak in 2008 at 4.6% but has remarkably decreased to 1.5% in 2019 [18, 19] with 55% of adults living with HIV on antiretroviral therapy and 42% of persons on antiretroviral therapy virally suppressed. The NDHS shows a 16%, 42%, and 23% prevalence of malaria among children less than five years of age in 2003, 2008, and 2018. Pending recent nationwide data, data from local studies showed malaria prevalence of 16.7% in south-eastern Nigeria [20, 21], 10.3% in northern Nigeria in 2020 [21], and 19.1% in western Nigeria [20, 22]. The utilization of long-lasting insecticide-treated nets (LITNs) has increased from 29.4% in 2010 to 52.2% in 2018 [23]. The review found increasing numbers of households with an improved source of drinking water and sanitation at 56% to 66% and 27% to 56% respectively from 2008 to 2018. However, the data in 2021 shows 71.5% of people have access to basic drinking water and 38.3% to basic sanitation [10].

#### Non-communicable diseases

The WHO non-communicable diseases (NCD) dashboard shows that 18% and 4% of Nigeria’s adult population have raised blood pressure in 2015 and raised blood glucose in 2014 [24]. In 2017, a nationwide survey reported 38.1% as the prevalence of hypertension [25] while an urban community in the north-western Nigeria reported 23.3% prevalence for DM in 2018 [26]. In 2016, the WHO NCD dashboard for Nigeria shows that tobacco smoking was 6%, 11% in men, and 1% among women [24]. This was slightly different from the values from the National Strategic Health Development Plan II of 13.5%, 5.6%, and 6% in 2003, 2008, and 2018 respectively [12, 16]. In 2019, only 8.7% of women in Nigeria had ever been screened for cervical cancer [27].

#### Service capacity, readiness, and access

Access to essential medicines in terms of availability in primary health care centres is 26% in 2019, worse in rural health centres [28]. Nigeria has 30 primary health centers per 100,000 population and 15 primary healthcare hospital bed densities per 1000 persons [12]. Concerning health security, available data on the International Health Regulations core capacity index shows an increase from 38% in 2011 to 66% in 2016 [29]. Available data on health providers- doctors and nurses/midwives are shown to be 38.9 (1:2572) and 148 (1:677) per 100,000 population [13] (Table 1).

**Table 1:** Health worker density by categories in Nigeria

Cadre	Number registered	Density/100,000 population	Ratio
Doctors	65,759	38.9	1:2,572
Dentists	3,129	1.9	1:54,056
Optometrist	2,676	1.6	1:63,207
Dispensing Optician	168	0.10	1:1,006,793
Nurses/Midwives	249,566	148	1:677
Dental Nurses	266	0.15	1:635,868
Radiographers	1,286	0.76	1:131,525
Pharmacists	16,979	10	1:9,961
Physiotherapists	2,818	1.7	1:60,022
Community Health Officers	5,986	3.5	1:28,256
SCHEW	42,938	25.3	1:3,939
JCHEWs	28,458	16.8	1:5,914
Medical Lab Scientists	19,225	11.3	1:8,798
Medical Lab Assistant	11,067	6.5	1:15,283
Medical Lab. Technicians	8,202	4.8	1:20,622
Environ. Health Officers	6,542	3.9	1:25,854
Health Records Officers	2,926	1.73	1:57,806
Dental Technologists	730	0.43	1:227,646
Dental Therapists	3,253	1.9	1:51,995
Dental Technicians	1,885	1.1	1:89,730
Dental Surgery Assistant	886	0.5	1:190,904
Health Technicians	8,739	5.15	1:19,354

Source: Adapted from NSHDP II (2018) and Health Workforce Profile, FMOH (2012)

### Equity in access to health services

#### Reproductive, maternal, new-born, and child health

**Fig. 6:**
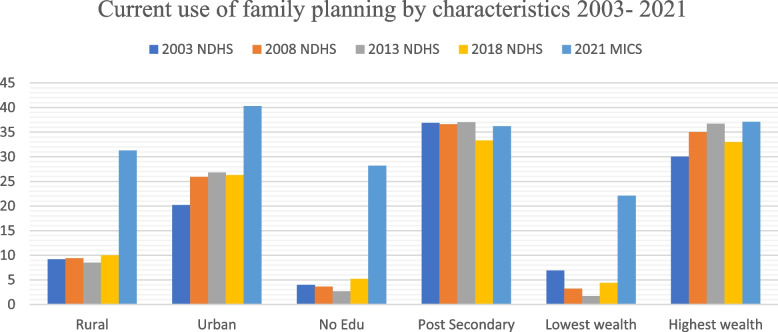
Trend in current use of family planning by socio-economic characteristics between 2003 and 2021 in Nigeria

Our findings show that over the years, (between 2003 and 2021), there has been a constant disparity in current use of family planning among rural/ urban dwellers, people of different educational levels and wealth indexes (Fig. 6) [7, 8, 30–32]. In 2018 for instance, the use of any contraceptive method is higher among currently married women in urban areas (26%) than among those in rural areas (10%) [4]. Similarly, the proportion of currently married women using modern contraceptive methods is higher among those with more than a secondary education (23%) than among those with no education (4%) [4]. While the percentage of currently married women using modern contraceptives increases with increasing household wealth, from 4% among those in the lowest wealth quintile to 22% among those in the highest quintile [4].

For all the markers of ANC- number of ANCs attended, skilled ANC attendant, health facility delivery, and delivery by skilled birth attendant among others, the inequity in accessing care in this dimension has remained since 2003. Between 2003 and 2021 the available data showed that the women who live in urban areas are two times more likely to have about 4 ANC visits than women who live in rural areas [7, 9].

In 2021, 84% of women living in the urban area received ANC from a skilled provider as opposed to 59% of women living in rural areas. Level of education also played a role in having a skilled provider of ANC service. About 48% of women with no education had ANC by a skilled provider while 96% of women with post-secondary had ANC by a skilled attendant [10]. Socioeconomic status is another indicator that describes inequity in receiving skilled ANC with women highest wealth quantile being twice like to have skilled ANC attendants as women in the lowest wealth quantile [10]. For facility delivery, rural women and women with no education were less likely to deliver at the facility than women living in the urban area and women with more than secondary education. With regards to delivery by a skilled birth attendant, geographical location, (living in an urban area), greater than secondary school education and high socioeconomic status are all positive correlates [7, 8, 30–32].

Concerning childhood immunization, the most recent National data showed that children living in urban areas are almost two times more likely than rural children to receive all basic vaccinations (52% versus 26%). Children whose mothers have more than a secondary education are more likely to be fully immunised than those born to mothers with no education (71% and 21% respectively). Similarly, 65% of children in the highest wealth quintile are fully immunised, as compared with 21% of children in the lowest wealth quintile [10].

#### Infectious disease control

Women and men in urban areas were more knowledgeable about TB than their counterparts in rural areas. Also, post-secondary education and high wealth quantile correlated positively with TB knowledge [7]. The utilization of TB treatment services shows that TB there is no disparity in the uptake of TB drugs in terms of different wealth indexes [33]. However, that higher percent of urban TB patients used some TB services including Gene-Xpert and X ray than those in rural areas. This implies that the urban dwellers received a higher share of gross and net benefits from TB services [33].

Having post-secondary-education and being wealthy was found to be an advantage in knowledge about AIDS. Urban dwellers’ post-secondary school education attainment and those that fall in the highest wealth quantile were found to have a more accepting attitude towards those living with HIV than their counterparts in the rural area, with no education and those in the lowest wealth quantile. Majority of people that tested for HIV lived in the urban area, had more than a secondary school education and had more income. A similar finding was obtained for the knowledge of the prevention of mother-to-child transmission of HIV [7].

**Fig. 7:**
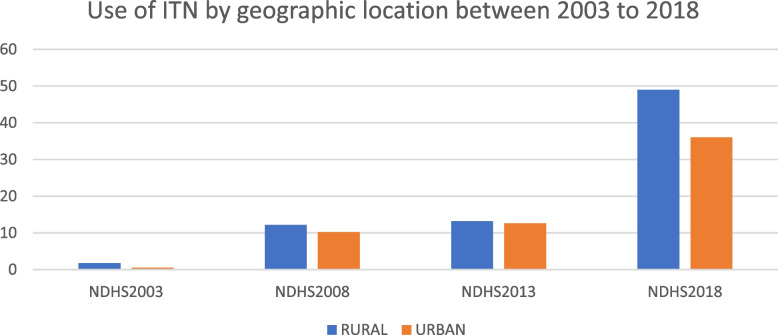
Use of ITN by geographic location between 2003 and 2018

The review also shows that from 2003 to 2018 (Fig. 7), more rural households own an insecticide treated net (ITN) than urban households. Rural dwellers were more likely to use an ITN than urban dwellers this was elicited by asking those who slept under an ITN the night before the survey. The review shows that over the years, households in the lowest wealth quantile were more likely to own an ITN than wealthier households [7].

**Fig. 8:**
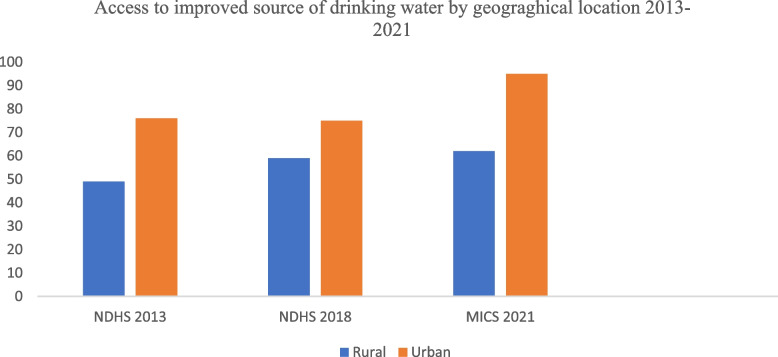
Trend in access to improved water source by location between 2013 and 2021in Nigeria

With regards to adequate sanitation, evidence shows that more urban households have improved toilet facilities than rural households while households in rural areas have more non-improved facilities and no facilities at all than the urban households [10]. Available data from 2013 to 2021, showed evidence of inequity of access to improved drinking water supply by geographical location (Fig. 8) [7, 8, 10].

#### Non-communicable diseases

In 2008, it was found that more men in rural areas smoked a cigar than those in urban areas. Less wealthy people smoked more than the highly wealthy [7] In 2018, educational level was found to be a determinant of the smoking habit. Individuals with a secondary education and above were found to smoke less than people with no education at all, 3% and 10% respectively [11].

#### Service capacity and access

Having health insurance positively correlated with the urban population, people with more than secondary education and in the highest wealth quantile [30]. The most recent Nigerian health strategic development plan reported gross and persistent mal-distribution of available health workers to the disadvantage of rural areas, and lower-level health facilities [27].

## Discussion

The study analyzed progress in service coverage and equity in access to healthcare within the Nigerian health systems based on the tracer indicators of the WHO framework for monitoring UHC. Generally, the progress observed over the period 2008–2018 was slow and not sufficient to achieve a minimum of 80% UHC by 2030. Our finding is similar to a report that Nigeria scored less than average (42%) in both 2015 and 2017 in the UHC service coverage index assessment [32]. This implies that, at this slow pace, Nigeria may not be able to attain UHC by 2030 if it does not intensify efforts toward addressing existing health service coverage gaps.

The slight progress in coverage of RMNCH tracer indicators may be attributable to the implementation of pockets of programs aimed at improving access to healthcare. However, this is still not sufficient as the health outcomes of RMNCH services show slow improvement over the years. For instance, the maternal mortality ratio was 545, 576, and 512 per 100,000 live births in 2008, 2013, and 2018 respectively [4]. Similarly, infant mortality reduced from 87 in 1990 to only 63 deaths per 1000 live births in 2021 while under-five mortality reduced from 193 to only 102 deaths per 1000 live births from 1990 to 2021 respectively [4]. To improve the coverage and possibly health outcomes of RMNCH, Nigeria could leverage effectively implementing the basic minimum health service package under the Basic Health Care Provision Fund (BHCPF) [34] and the Task Shifting and Task Sharing Policy which empowers community pharmacists and patent medicine vendors to administer modern contraceptive methods [34].

Our findings on low skilled birth attendance (SBA) agree with a study in Nigeria which reported that the number of antenatal care (ANC) services were positively associated with having SBA during childbirth and not the number of ANC visits attended [35]. In addition, some studies showed that the availability of skilled health workers and access to the health facility positively influence the utilization of SBAs [36, 37]. The increasing numbers attending ANC visits and using SBA may most likely contribute to the slowly dropping maternal mortality rates.

The improvement in the health-seeking behaviour for acute respiratory infections corresponds with the decrease in under-five mortality per 100 live births from 201 in 2003 to 132 in 2018. The NDHS vaccination data differs from a study where 22.1% were fully vaccinated and 29% of children had never been vaccinated [38]. The reasons for the vaccination numbers are listed by this study as immunization coverage was significantly associated with childbirth order, delivery place, child number, and presence or absence of a child health card. Maternal age, geographical location, education, religion, literacy, wealth index, marital status, and occupation were significantly associated with immunization coverage. Paternal education, occupation, and age were also significantly associated with coverage. Respondent's age, educational attainment and wealth index remained significantly related to immunization coverage at a 95% confidence interval in multivariate analysis [38].

Governance in the tuberculosis control program in Nigeria has been proffered as the reason for the abysmally slow progress in the tuberculosis program as seen in our findings. Some governance issues implicated are low public spending on TB control, health workers not being transparent in communicating service entitlements to users, weak integration of TB control into the community and general health services, weak drug supply system, and TB surveillance [39].

The reduced prevalence of HIV to 1.5% in 2019 in Nigeria shows some in Nigeria although the UNAIDS 90–90-90 goal for 2020 [40] was not met. We showed that 42%, not 90% of persons on antiretroviral drugs were virally suppressed. Etsetowaghan explained that the prevalence among key populations is still high and would sabotage the UNAIDS goals attainment in Nigeria [41]. The factors that affect the progress on HIV/AIDS in Nigeria include poor technical management of HIV/AIDS services, lack of country ownership, ineffective supply chain management systems, lack of a sustainability plan, disconnect in integrating HIV/AIDS services into the Nigerian health system and lack of coordination among the multiple actors who often work in silos [42, 43].

The picture of malaria prevalence in under-fives is encouraging with a drop from 42% in 2008 to 16.7% in 2021. However, Nigeria and 10 other countries still contribute significantly to the proportion of malaria cases and deaths in high-burden countries in the Africa Region according to a report by WHO-AFRO in 2021 [44] to achieve the UHC for malaria requires targeted approaches to drive down malaria cases and death more quickly through a multi-sector coordinated national response, implementation of strategic health plans and strategies for malaria guided by local evidence and financing and resource mobilization to increase malaria awareness, through targeted communication and active community participation.

Our findings on the prevalence of non-communicable diseases conform with the report of WHO [44]. The high prevalence of NCD in this study could be attributed to lifestyle modification including tabacco use, harmful use of alcohol, physical inactivity, and unhealth diets. The Nigerian government has a national policy and strategic plan of action on NCDs [45] which when effectively implemented, could reduce the high prevalence of NCDs.

There is evidence that access to basic health services and health outcomes are unevenly distributed across different social groups [6]. This inequality is largely tied to disparities in educational level, socioeconomic status and geographic locations of the population.

Our findings show that knowledge of contraceptive methods is lowest among women with no education, in the lowest wealth quintile and women who live in rural areas. The same holds for use of contraceptives. This inequality has been consistent from 2003 till date. Adeleye and Adeleye in 2003, used education to determine the knowledge attitude and practice of contraceptives in Ibadan, and they found that knowledge and use of contraceptives were positively related to education [46]. The inequity in service utilization cuts across most low- and middle-income countries and evidence abounds that poor people are more likely to forgo health care than their wealthier counterparts. Equally service utilization is determined by rural–urban location [47–49]. In the bid to improve the inequality in service delivery for achieving UHC, the private sector engagement became apparent, yet the same pattern of delivery and outcomes was noted. In 2015 indices in Low- and middle-income countries showed that 25% of people in the lowest wealth quantile accessed family planning service in the private sector while up to half of the individuals in the highest wealth quantile accessed private sector FP services [50].

The inequity in accessing care with regard to antenatal and delivery has been and is still an ongoing challenge in Nigerian healthcare utilization. Women’s level of education informed accessing ANC care by skilled birth attendants as well as their place of residence. The more educated a woman is, the more knowledgeable the individual is on the benefits and risks of accessing appropriate health care. Another indicator that describes inequality in receiving skilled ANC is socio-economic status and it has been shown that women in the highest wealth quantile were two times more likely to have skilled ANC than women in the lowest quantile. This simply shows that the wealth of pregnant women influences their health in positive ways. The wealthier a woman, the more likely she is to be educated, have more knowledge about ANC and the more likely to live in a developed area [51].

The level of poverty in Nigeria has a role to play in the socioeconomic inequality in utilizing health care services. The implication of the correlation between wealth and ANC attendance is that it is associated with the cost of purchase of drugs, hospital/ consultation fees and other indirect costs such as transportation costs [51]. hence, the objective of the health insurance scheme is to alleviate the financial stress on the poorer households with regards to healthcare utilization but on the contrary, the richer group appear to be benefiting more because of the low coverage of the scheme [51]. The decentralization of the social health insurance scheme is in the right direction to improve coverage of quality and equitable access to healthcare across Nigerian states and may facilitate the attainment of UHC.

To take care of the inequality in maternal health care utilization, David-Wayas et al. in their article published in 2017 made some recommendations which include increasing efforts that target utilization in rural areas by emphasizing the benefits of modern ANC in churches, mosques and other places of worship. She also suggested that education of the woman should be strongly encouraged by the government because this will have a positive effect on ANC utilization as well as reduce observed inequality in healthcare utilization in the long run [51]. Childhood immunizations have also shown the same pattern where a mother’s educational level, area of residence and wealth status determines the chances of a child being fully immunized. The mentioned barriers also pose problems in intervening to improve immunization uptake. While designing an Immunization Reminder and Information SMS System (IRISS), a survey carried out to study the factors that would inform design and implementation in Kebbi State Nigeria found that most people in the communities of interest did not own phones, those that possessed phones did not know how to open SMS messages and could not read [52].

The factors perpetuating inequality in service utilization as seen above cut across other tracer indicators of service delivery and utilization like control of infectious diseases, and communicable diseases. The only inverse scenario to what we have seen all along is in the case of cigar smoking where men who lived in the rural area were found to smoke more than men in the urban area, [25] and those with no education were found to smoke more than people who have post-secondary education [25]. This is most likely because the situation being reviewed is a negative one and those with no education who live more in the rural areas are more likely to fall prey because of a lack of knowledge.

The developed world has beyond health insurance, come up with other strategies of tackling health inequalities. The European Union (EU) is one such community. To address health inequalities, they decided to approach it by handling the social determinants of health. Their parliament emphasized that health inequalities are cause by social inequalities in terms of living conditions and models of social behaviour linked to employment and unequal distribution of medical assistance and income, education, gender, race health promotion services etc [32]. They proposed supporting the action on health inequalities through various EU funds like the Fund for European Aid to the most Deprived (FEAD) to alleviate the worst forms of poverty by providing social inclusion activities, material assistance and food support [32]. Another form of EU funding support is the European Social Fund (ESF) that provides co-funding to actions aimed at helping people access the labour market, and at improving the situation of the most vulnerable [32]. Nigeria being a developing nation might not have all the resources to provide extra funding but it could adapt this measure in a way that it can and this will possibly go a long way in improving health inequality in health care service delivery.

## Conclusion

The Nigerian service delivery progress towards UHC leaves sufficient room for improvement despite the slow strides made in some areas. To achieve UHC as well as health-related SDGs, the delivery of health services will need to address underlying issues that cause inequalities in health like the social determinants of health, governance issues and corruption amongst others. There is a need for a concerted effort across all tiers of government and more investment in health service delivery to facilitate improvement in health outcomes as well as a need to leverage on the country’s decentralized system and to make policies and design programs that will improve the living conditions and social behaviour linked to employment and equal distribution of medical assistance and income, education, gender, race, health promotion services. Adopting some of the suggested intervention/approaches in the discussion section could help in improving health services coverage in an equitable manner towards achieving UHC in Nigeria.

## Data Availability

The datasets used and/or analysed during the current study available from the corresponding author on reasonable request.

## Abbreviations

ANC: Ante natal care
LITNs: Long-lasting insecticide-treated bed nets
NCD: Non-communicable diseases
NDHS: National demographic health survey
RMNCH: Reproductive, maternal, newborn, and child health
SBA: Skilled birth attendant
SDGs: Sustainable Development Goals
TB: Tuberculosis
UHC: Universal Health Coverage
WHO: World Health Organization
